# Supplementary material for: Transport of DNA within cohesin involves clamping on top of engaged heads by Scc2 and entrapment within the ring by Scc3
Source: eLife. 2020 Sep 15;9:e59560. doi: 10.7554/eLife.59560 (PMC7492086; doi:10.7554/eLife.59560)
Supplement: Supplementary file 1. [file elife-59560-supp1.docx]

|  | **+ 40 bp DNA** | | **+ circular DNA** |
| --- | --- | --- | --- |
| ***S. cerevisiae* cohesin** | **Tetramer**  Smc1E1158Q, Smc3E1155Q, Scc1, Scc2C2, ATP | **Tetramer head segment**  Smc1E1158Q,  Smc3E1155Q, Scc1,  Scc2C2, ATP  (EMD-11585, PDB 6ZZ6) | **T. head segment**  Smc1E1158Q, Smc3E1155Q, Scc1, Scc2C2, ATP |
| **Data collection and processing** |  |  |  |
| Magnification | 81,000 | 81,000 | 105,000 |
| Voltage (kV) | 300 | 300 | 300 |
| Electron exposure (e–/Å^2^) | 55 | 55 | 40 |
| Defocus range (μm) | 1.5 ~ 3.3 | 1.5 ~ 3.3 | 0.6 ~ 1.0 |
| Pixel size (Å) | 1.07 | 1.07 | 1.0 |
| Symmetry imposed | *C1* | *C1* | *C1* |
| Initial particle images (no.) | 1,516,413 | 2,314,881 | 65,442 |
| Final particle images (no.) | 21,343 | 588,164 | 23,728 |
| Map resolution (Å)  FSC threshold | 10  0.143 | 3.35  0.143 | 7.3  0.143 |
| Map resolution range (Å) | 10 – 50 | 3.2 – 50 | 7.3 – 50 |
|  |  |  |  |
| **Refinement** |  |  |  |
| Initial model used (PDB) |  | 1W1W, 4UX3, 5ME3 |  |
| Model resolution (Å)  FSC threshold |  | 3.35  0.143 |  |
| Model resolution range (Å) |  | 3.2 – 4.5 |  |
| Map sharpening *B* factor (Å^2^) |  | -88.25 |  |
| Model composition  Non-hydrogen atoms  Protein residues  Nucleotide residues  Ligands |  | 16591  1916  Smc1(2-71, 87-195, 1044-1224); Smc3(1-228, 997-1071, 1104-1222); Scc1(67-103, 502-510, 519-555); Scc2(221-236, 250-262, 278-291, 304-322, 336-373, 386-589, 597-634, 647-662, 678-617, 927-1049, 1059-1078, 1090-1184, 1203-1342, 1355-1398, 1413-1434, 1448-1456, 1466-1475)  68 (poly-A)  2 ATP, 2 Mg |  |
| *B* factors (Å^2^)  Protein  Nucleotide  Ligand |  | 15.18  72.00  21.89 |  |
| R.m.s. deviations  Bond lengths (Å)  Bond angles (°) |  | 0.003  0.571 |  |
| Validation  MolProbity score  Clashscore  Poor rotamers (%) |  | 1.77 (86^th^ percentile)  7.92  0.00 |  |
| Ramachandran plot  Favored (%)  Allowed (%)  Disallowed (%) |  | 95.05  4.95  0.00 |  |
